# Altered protein expression in serum from endometrial hyperplasia and carcinoma patients

**DOI:** 10.1186/1756-8722-4-15

**Published:** 2011-04-14

**Authors:** Yi-sheng Wang, Rui Cao, Hong Jin, Yi-ping Huang, Xiao-yan Zhang, Qing Cong, Yi-feng He, Cong-jian Xu

**Affiliations:** 1 Department of Gynecology, Obstetrics and Gynecology Hospital, Fudan University, 419 Fangxie Road, ShangHai, China; 2 DaLian Obstetrics and Gynecology Hospital, 1 Dunhuang Road, DaLian, China; 3 Institutes of Biomedical Sciences, Fudan University, 138 Medical College Road, ShangHai, China; 4 Department of Chemistry, Fudan University, 220 Handan Road, ShangHai, China; 5 Department of Obstetrics and Gynecology, ShangHai Medical College, Fudan University, 138 Medical College Road, ShangHai, China; 6 Key Laboratory for Disease Related to Women's Reproduction and Endocrine System, 413 Zhaozhou Road, ShangHai, China

## Abstract

**Background:**

Endometrial carcinoma is one of the most common gynecological malignancies in women. The diagnosis of the disease at early or premalignant stages is crucial for the patient's prognosis. To date, diagnosis and follow-up of endometrial carcinoma and hyperplasia require invasive procedures. Therefore, there is considerable demand for the identification of biomarkers to allow non-invasive detection of these conditions.

**Methods:**

In this study, we performed a quantitative proteomics analysis on serum samples from simple endometrial hyperplasia, complex endometrial hyperplasia, atypical endometrial hyperplasia, and endometrial carcinoma patients, as well as healthy women. Serum samples were first depleted of high-abundance proteins, labeled with isobaric tags (iTRAQ™), and then analyzed via two-dimensional liquid chromatography and tandem mass spectrometry. Protein identification and quantitation information were acquired by comparing the mass spectrometry data against the International Protein Index Database using ProteinPilot software. Bioinformatics annotation of identified proteins was performed by searching against the PANTHER database.

**Results:**

In total, 74 proteins were identified and quantified in serum samples from endometrial lesion patients and healthy women. Using a 1.6-fold change as the benchmark, 12 proteins showed significantly altered expression levels in at least one disease group compared with healthy women. Among them, 7 proteins were found, for the first time, to be differentially expressed in atypical endometrial hyperplasia. These proteins are orosomucoid 1, haptoglobin, SERPINC 1, alpha-1-antichymotrypsin, apolipoprotein A-IV, inter-alpha-trypsin inhibitor heavy chain H4, and histidine-rich glycoprotein.

**Conclusions:**

The differentially expressed proteins we discovered in this study may serve as biomarkers in the diagnosis and follow-up of endometrial hyperplasia and endometrial carcinoma.

## Background

Endometrial carcinoma (ECa) is one of the most common gynecological malignancies in women. During the past two decades, the incidence of ECa in China has been increasing consistently [1]. Endometrioid ECa, the predominant subtype of ECa, is preceded by a series of precursor lesions that include simple endometrial hyperplasia (SEH), complex endometrial hyperplasia (CEH), and atypical endometrial hyperplasia (AEH). To reduce the incidence of ECa, it is preferred to diagnose and treat patients at the stages of the various endometrial hyperplasias before progression to ECa. Unfortunately, examining the severity of endometrial lesions requires invasive tissue sampling procedures [2], such as dilation and curettage. So far, no facile and non-invasive test exists for both the diagnosis and surveillance of endometrial hyperplasia (EH) and ECa. The discovery of changes in protein profiles that correlate with the severity of endometrial lesions and can thus be used as biomarkers for the non-invasive diagnosis of endometrial hyperplasia and carcinoma is thus highly desirable.

Cancer formation is accompanied by a series of protein expression change in serum and cancerous tissues [3]. A significant number of proteomics studies have been reported in which tissue and/or blood samples from ECa patients have been analyzed [4-17]. However, most of these studies only compared samples between cancer patients and healthy women, and thus lacked the critical information on disease progression that can be provided by directly analyzing endometrial hyperplasia samples. The only proteomics investigation that has focused on endometrial hyperplasia identified several proteins with altered expression exclusively in resected endometrial hyperplasia tissue [12]. However, biomarker candidates discovered from tissue samples need to be further evaluated in body fluids (*e.g*. blood and urine) that can be used more practically for diagnosis.

Clinical biomarker discovery using proteomic approaches has been limited by a relatively high variation in sample preparation techniques and by the low reproducibility of quantitative measurement using mass spectrometry (MS). The development of isobaric tags for relative and absolute quantification (iTRAQ), which allows simultaneous measurement of multiple (up to 8) samples in one experimental run, significantly reduces the potential variation in multiple MS runs, and thus improves the accuracy of protein identification and quantification [18]. The iTRAQ technology has been successfully applied to biomarker discovery for many conditions in both tissue [4] and serum samples [19].

In this study, we reported a quantitative proteomics analysis using the iTRAQ technology to investigate protein changes in serum during the multiple stages of disease progression in ECa. With the iTRAQ technology, we specifically compared serum samples from multiple stages of hyperplasias (SEH, CEH, and AEH) and ECa. We found several proteins with altered expression levels during disease progression that could represent serum biomarker candidates in EH and ECa.

## Results and discussion

In this study, iTRAQ technology in combination with 2D LC-MS/MS was applied to detect differentially expressed proteins in EH and ECa. Serum samples from 20 patients (6 patients of SEH, 4 of CEH, 4 of AEH, and 6 of stage I endometrioid ECa) and 7 healthy women who were free of metabolic disorders were used. Although expression of serum high-abundance proteins were reported to show stage correlative changes in some malignant conditions [20], we applied a serum depletion procedure (see Materials and Methods for details) in this study to deplete the high-abundance proteins that could interfere with the detection of low-abundance proteins of greater biological interest. Proteins from depleted serum samples were digested into peptides, individually labeled with iTRAQ reagents, combined, and subjected to LC-MS/MS analysis.

This iTRAQ-based proteomics analysis led to the identification of a total of 15209 peptides, 3766 of which were unique. These identified peptides correspond to a set of 430 proteins with more than 95% confidence (ProtScore > = 1.3). Among them, 74 non-redundant proteins were successfully quantified with average ratios presented. The iTRAQ ratios were calculated over the control samples from normal individuals (iTRAQ channel 117). Because we applied the depletion procedure to remove the high-abundanc proteins, these proteins were not included in further data analyses.

**Figure 1:**
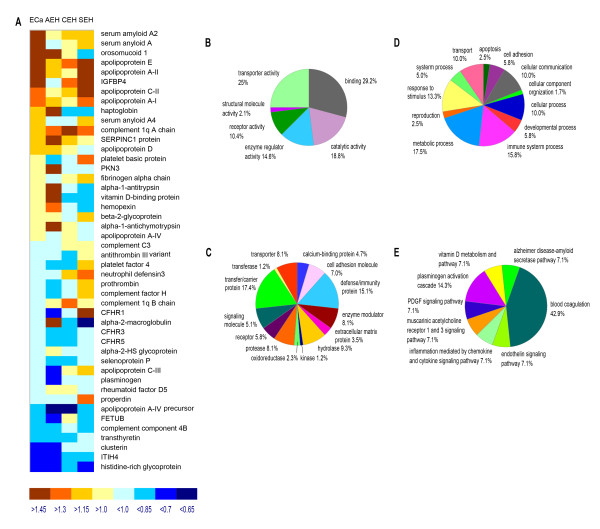
**Overview of protein identification and quantitation results**. **(A) **Average ratio of proteins in SEH, CEH, AEH, and ECa groups. **(B) **PANTHER analysis for molecular function, **(C) **protein class, **(D) **biological process, and **(E) **pathway of identified proteins.

**Figure 2:**
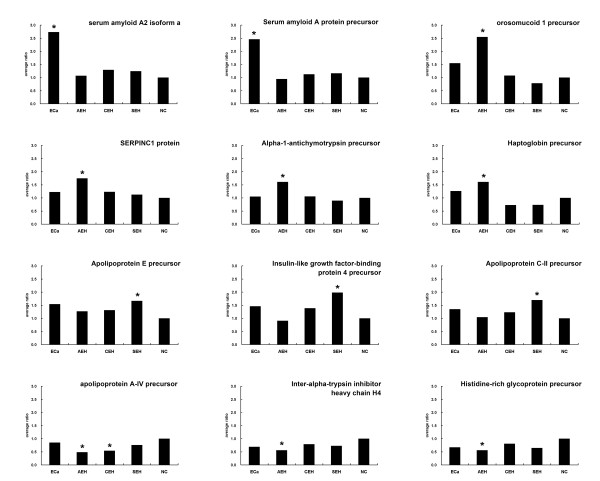
**Expression profiles of 12 proteins with significant changes in endometrial hyperplasia or carcinoma**. (*), Expression change greater than 1.6-fold, *i.e*. average ratio >1.6 or <0.625, when compared with normal control.

An overview of the resulting set of proteins is shown in Figure 1. The majority of proteins do not appear to be ECa-related because their expression levels show no linear correlation with the disease progression (Figure 1A). Gene Ontology analysis indicated that these proteins are primarily constitutional serum proteins involved in typical blood pathways including transport, immune response, or blood coagulation (Figure 1B-1E). However, we did identify several proteins whose expression levels were significantly increased or decreased among the stages of EH and ECa (Figure 2)

Using a 1.6-fold quantification cutoff for those proteins with a relatively significant change, 12 proteins quantified at least once in the four disease groups show significant changes in their expression and were followed as potential cancer markers (Figure 2 and Table 1). Four of these proteins, including serum amyloid A (SAA), apolipoprotein A-IV (ApoA4), antithrombin III (synonymous with SERPINC1), and inter-alpha-trypsin inhibitor heavy chain H4 (ITIH4; synonymous with inter-alpha-trypsin inhibitor family heavy chain-related protein, IHRP), have been reported previously (Table 2). Our detection of SAA, ApoA4, and antithrombin III is consistent with previous reports, while the opposite result has been observed for ITIH4 [6,16,21].

**Table 1:** List of proteins identified as potential cancer markers in the serum of endometrial hyperplasia and carcinoma patients.

N	%Cov	Accession	Protein Name (Gene Symbol)
1	42.19	IPI00550991	alpha-1-antichymotrypsin precursor (SERPINA3)
2	55.98	IPI00844156	antithrombin III(SERPINC1)
3	90.40	IPI00304273	apolipoprotein A-IV precursor (APOA4)
4	94.06	IPI00021856	apolipoprotein C-II precursor (APOC2)
5	64.98	IPI00021842	apolipoprotein E precursor (APOE)
6	68.57	IPI00641737	haptoglobin precursor (HP)
7	71.05	IPI00022371	histidine-rich glycoprotein precursor (HRG)
8	25.58	IPI00305380	insulin-like growth factor-binding protein 4 precursor (IGFBP4)
9	57.78	IPI00218192	inter-alpha-trypsin inhibitor heavy chain H4 (ITIH4)
10	42.29	IPI00884926	orosomucoid 1 precursor (ORM1)
11	99.18	IPI00552578	serum amyloid A protein precursor (SAA1;SAA2)
12	100.00	IPI00006146	serum amyloid A2 isoform a (SAA1;SAA2)

**Table 2:** Potential cancer markers for endometrial hyperplasia and carcinoma reported in previous literatures.

Protein Name	Endometrial Carcinoma		Endometrial Hyperplasia	
	
	Tissue	Serum/Plasma	Tissue	Serum/Plasma
alpha-1-antitrypsin		-[6]		
alpha-1-antitrypsin precursor	-[4]			
alpha-1-beta glycoprotein		+[6]		
alpha-enolase			+[12]	
antithrombin III		+[6]*		
apolipoprotein A-IV		-[16]*		
calcyphosine	+[14]			
calgizzarin	+[4]			
calgranulin A	+[11]			
cAMP dependent protein kinase type I-beta regulatory chain	+[12]			
chaperonin 10	+[4,7,11]			
cleaved high molecular weight kininogen		-[4,6]		
clusterin		+[6]		
complement component 3		+[16]		
complement component 4A		+[16]		
complement component 4B		+[16]		
creatine kinase B	-[4]			
cyclophilin A	+[14,17]			
epidermal fatty acid binding protein	+[14]			
GAPDH	+[12]			
heat shock 27 kDa protein	+[12]			
heat shock 70 kDa protein 1	+[12]		+[12]	
heat shock cognate 71 kDa protein	+[12]		+[12]	
heterogeneous nuclear ribonucleoprotein D0	+[4]			
heterogeneous nuclear ribonucleoproteins A2/B1	+[12]			
inter-alpha-trypsin inhibitor family heavy chain-related protein (IHRP)		+[6,16,21]^#^		
leucine-rich glycoprotein		+[6]		
macrophage migratory inhibitory factor	+[4]			
phosphoglycerate kinase	+[12]		+[12]	
polymeric immunoglobulin receptor precursor	+[4]			
prohibitin	+[12]			
prolactin		+[15]		
pyruvate kinase M1 or M2 isozyme	+[4]			
serotransferrin precursor	+[12]			
serum albumin precursor	+[12]		+[12]	
serum amyloid A		+[15]*		
transgelin	-[4]			
trypomyosin fibroblast isoform TM3	+[12]			
vimentin			+[12]	

References are indicated in brackets;

"+", up-regulation;

"-", down-regulation;

"*", consistent result in this study when compared with previous studies;

"#", contradictory result in this study when compared with previous studies.

ITIH4 protein is a 120KD glycoprotein, which is prone to be cleaved to produce fragments of different length [16]. In the previous studies, serum level of ITIH4 in ECa patients was reported to be upregulated [6]. After MS analysis, these ITIH4 were identified as 35KD fragment of the whole ITIH4 protein [16,21]. In this study, iTRAQ method is unable to differentiate cleaved fragments from whole protein. All fragments encoded by ITIH4 gene were used for ITIH4 quantitation. This may be the basis of the contradictory result and low confidence of quantitation (*p *= 0.09) in this study.

Two proteins, serum amyloid A protein precursor and serum amyloid A2 isoform α, showed significant elevation in ECa as compared with the normal control. Intermediate upregulation of these two proteins was also observed in the serum samples from AEH, CEH, and SEH (Figure 2). SAA proteins belong to a family of apolipoproteins that are synthesized mainly in the liver in response to inflammatory stimuli as acute-phase proteins [22]. The expression levels of these proteins in serum have been found to increase in a broad spectrum of neoplastic diseases, and high levels have been positively correlated with metastasis and poor prognosis [23]. A study in colon carcinoma has demonstrated gradually increased expression of SAA as epithelial cells progress from dysplasia to neoplasia, suggesting that this protein plays a role in colonic tumorigenesis [24]. Previous proteomic analyses of ECa tissues did not observed significantly altered expression of SAA in cancerous tissue [4,7,8,10,25]. However, downregulation of the SAA2 gene has been observed in one study using micro-dissected endometrioid endometrial carcinoma tissues [26]. Thus, it remains to be determined whether the elevation of SAA levels in the serum of ECa patients originates from liver secretion or from endometrial cancerous tissues.

Three additional proteins, apolipoprotein C-II precursor, apolipoprotein E precursor, and apolipoprotein A-IV precursor, showed consistently altered expression with high confidence levels in the four disease groups (Figure 2). Upregulation of apolipoprotein C-II precursor and apolipoprotein E precursor in SEH and downregulation of apolipoprotein A-IV precursor in CEH and AEH were of significance according to the given benchmark. Patients with EH and ECa also usually have the complication of a lipid metabolism disorder. In the present study, all participants were free of hyperlipoidemia at enrollment, and serum samples were collected after a fasting period of more than 8 hours. However, abnormal apolipoprotein levels still presented. This result may imply a systemic impairment of lipid metabolism in EH and ECa patients.

Histidine-rich glycoprotein (HRG) precursor was downregulated in the four disease groups, with a ratio over the benchmark only in atypical hyperplasia (Figure 2). HRG is a member of the cystatin superfamily. A study of HRG-knockout mice has suggested a property of mild anti-coagulant and anti-fibrinolytic activity of HGR *in vivo *[27]. Other properties of HRG, such as antibacterial activity [28], have also been reported. HRG was found to exert anti-tumor effects *in vivo *through the inhibition of tumor vascularization [29]. Although downregulation of HRG reached the benchmark only in atypical hyperplasia in the present study, this result may suggest a propensity for patients to progress to ECa.

Haptoglobin (HP) precursor was upregulated in AEH and ECa, but downregulated in CEH and SEH with high confidence (Figure 2). An elevated serum concentration of this protein has been associated with several malignant diseases, such as lung cancer [30] and cervical cancer [31]. One recent report on HP expression levels in endometrioid adenocarcinoma tissue has reported a general upregulation of mRNA and protein levels of HP in both cancerous and adjacent non-affected endometrial tissues [32]. These data suggest that endometrial tissue can be one of the origins, though not the only one, responsible for elevated serum HP levels in ECa patients.

Insulin-like growth factor-binding protein 4 precursor (IGFBP-4) was upregulated significantly in SEH and to a mild extent in CEH and ECa (Figure 2). The relationship between the serum level of IGFBP and ECa risk remains controversial [33,34]. The relationship between the expression of IGFBP-1, IGFBP-2, and IGFBP-3 with endometrial carcinoma has been frequently investigated. Little is known about IGFBP4.

## Conclusions

In conclusion, we conducted a serum proteomic analysis of endometrial hyperplasia and carcinoma using iTRAQ technology and 2D LC-MS/MS. In addition to the upregulation of SAA in ECa, we report for the first time the altered expression level of 7 proteins in AEH. These proteins may serve as potential biomarkers for the early diagnosis and surveillance of endometrial carcinoma and hyperplasia.

## Methods

### Samples

This study was approved by the institutional review boards of the Obstetrics and Gynecology Hospital, Fudan University, Shanghai, P.R. China. All participants provided written informed consent at enrollment. For proteomic analysis, untreated, pathologically confirmed EH and stage I endometrioid ECa patients were enrolled in this study from May 2007 to February 2009. Healthy women undergoing routine physical examinations were recruited as normal controls (NC) during the same period. Because metabolic disorders, such as hypertension, diabetes mellitus, and hyperlipoidemia, result in obvious changes in protein expression in serum [35], all participants with these disorders were excluded from this study. Ultimately, 20 patients with endometrial lesions (including 6 SEH, 4 CEH, 4 AEH, and 6 stage I endometrioid ECa) and 7 healthy women were enrolled. The median ages at diagnosis were 46 years (range 43 to 52), 40 years (range 28 to 46), 33 years (range 29 to 40), and 53 years (range 44 to 62) for SEH, CEH AEH, and ECa patients, respectively. The median age of NCs was 46 years (range 45 to 47). Four women in the ECa group, 1 in the SEH group, none in the CEH group, and 1 in the NC group were postmenopausal. Five milliliters of blood samples were taken from each participant. After clotting and centrifuging at 2000 rpm for 10 min, the serum was stored at -80°C until use.

### Depletion of high-abundance proteins

Serum samples were thawed on ice. Equal amounts of serum from individuals in each group were pooled to yield 5 distinct pools of 600 μl each. High-abundance proteins of each serum pool were depleted using ProteoMiner Protein Enrichment Kits (Bio-Rad, USA) according to the manufacture's instruction. Briefly, serum was loaded onto the column and proteins bound with high specificity to a bead-based library of diverse peptide ligands. High-abundance proteins which saturated their corresponding ligands were washed out of the column. The remaining low- and mid-abundance proteins in the column were then eluted and collected. The eluents were precipitated using a Ready Prep 2-D Cleanup Kit (Bio-Rad, USA). The total protein concentrations were determined by a Bradford protein assay as previously described [36].

### iTRAQ reagent labelling

After high-abundance protein depletion and concentration measurements, aliquots of 100 μg protein from each of the 5 sample pools were reduced, blocked on cysteines, and digested overnight at 37°C with trypsin, as described in the iTRAQ protocol. Peptides were then labeled individually with one iTRAQ tag (Applied Biosystems, USA) as follows: ECa, 113.1; SEH, 114.1; CEH, 115.1; AEH, 116.1; NC, 117.1. The labeled peptides were then pooled and dried using a rotary vacuum concentrator (Christ RVC 2-25, Christ, Germany).

### Strong cation exchange chromatography (SCX)

Strong cation exchange chromatography was performed on the ACQUITY Ultra Performance LC system (Waters, USA). Tryptic-digested and labeled peptides were loaded onto a 0.5 × 23 mm, 5 μm, 300 Å Column (Waters, USA) and eluted stepwise by injecting salt plugs of 10 different molar concentrations of 25, 50, 75, 100, 150, 200, 300, 400, 500, and 1000 mM NH_4_AC. Ten fractions were collected from the SCX column.

### LC-MS/MS

Fractions from the SCX column were analyzed on a Qstar XL LC/MS/MS system (Applied Biosystems, USA). Each fraction was loaded onto a ZORBAX 300SB-C18 reverse phase (RP) column (5 μm, 300 Å, 4.6 × 50 mm, Agilent, USA). Buffer A was composed of 5% acetonitrile, 95% water, and 0.1% formic acid, and Buffer B was composed of 95% acetonitrile, 5% water, and 0.1% formic acid. The elution was performed using a gradient ranging from 5% to 45% Buffer B at a flow rate of 0.4 μl/min for 90 min. The LC eluent was directed to a nano-flow electrospray source for MS/MS analysis in an information dependent acquisition mode. A TOF MS survey scan was acquired from 400-1800 m/z, with up to the 6 most intense multiply charged ions in the survey scan sequentially selected for MS/MS analysis. Product ion spectra were accumulated for 2 s in the mass range 100-2000 m/z with a modified Enhance All mode Q2 transition setting favoring low mass ions, so the reporting iTRAQ ion (113.1, 114.1, 115.1, 116.1, and 117.1 m/z) intensities were enhanced for quantitation. Each fraction from SCX chromatography was analyzed in duplicate.

### Protein identification and relative quantitation

MS/MS data was searched against the International Protein Index (IPI) database (version 3.45, HUMAN) using ProteinPilot™ software (version 2.0, Applied Biosystems, USA) with trypsin set as the digestion enzyme and methyl methanethiosulfonate as the cysteine modification. The search results were further processed by ProteinPilot™ software using the ProGroup Algorithm for redundant hits removing and comparative quantitation, resulting in the minimal set of justifiable identified proteins. Proteins with more than 95% confidence (ProtScore > = 1.3) were reported. Relative quantitation of peptides was calculated as a ratio by dividing the iTRAQ reporter intensity at 113.1, 114.1, 115.1, and 116.1 m/z by that at 117.1 m/z. The quantitation results were normalized for loading error among the 5 groups by bias correction calculated automatically by the ProteinPilot™ software. The ratios of peptides that support the existence of one protein were averaged for protein relative quantitation. A *p*-value was reported after one sample *t*-test of averaged protein ratio against 1 to assess the validity of the protein expression change. Protein ratios with a *p-*value less than 0.05 were considered reliable. Standard deviations (SD) of the protein ratio, which stem from technical variation, were reported to be less than 0.3 in 90% of iTRAQ experimental runs [37]. Therefore, we used a difference of 2 SDs, *ie*. protein ratio greater than 1.6 or smaller than 0.625, as an approximate benchmark for variation in protein expression. Expression changes greater than 1.6-fold in normalized expression levels were considered to be outside the range of technical variability.

### PANTHER analysis

The molecular function, protein classification, biological process and signaling pathway of proteins identified in this study were elucidated by searching against the PANTHER database. (http://www.pantherdb.org).

## List of Abbreviations

AEH: atypical endometrial hyperplasia; CEH: complex endometrial hyperplasia; ECa: endometrial carcinoma; HP: haptoglobin; HRG: histidine-rich glycoprotein; IGFBP-4: insulin-like growth factor-binding protein 4; IHRP: inter-alpha-trypsin inhibitor family heavy chain-related protein; IPI: international Protein Index; ITIH4: inter-alpha-trypsin inhibitor heavy chain H4; iTRAQ: isobaric tags for relative and absolute quantification; LC: liquid chromatography; MS/MS: tandem mass spectrometry; NC: normal control; SHE: simple endometrial hyperplasia; SAA: serum amyloid A; SCX: strong cation exchange chromatography; SD: standard deviation.

## Competing interests

The authors declare that they have no competing interests.

## Authors' contributions

YSW drafted the manuscript, participated in the study design and sample collection, and carried out data analysis. RC participated in the study design, patient enrolment, and sample collection. HJ carried out the high-abundance protein depletion, iTRAQ labelling, and LC/MS analysis. YPH participated in the sample collection and data analysis. XYZ participated in the study design and data analysis. QC participated in the study design and revised the manuscript. YFH participated in the LC/MS analysis and data analysis. CJX conceived of the study and participated in its design. All authors read and approved the final manuscript.
